# Supplementary figures and images for: Mixed Wolbachia infections resolve rapidly during in vitro evolution
Source: PLoS Pathog. 2024 Jul 25;20(7):e1012149. doi: 10.1371/journal.ppat.1012149 (PMC11302900; doi:10.1371/journal.ppat.1012149)

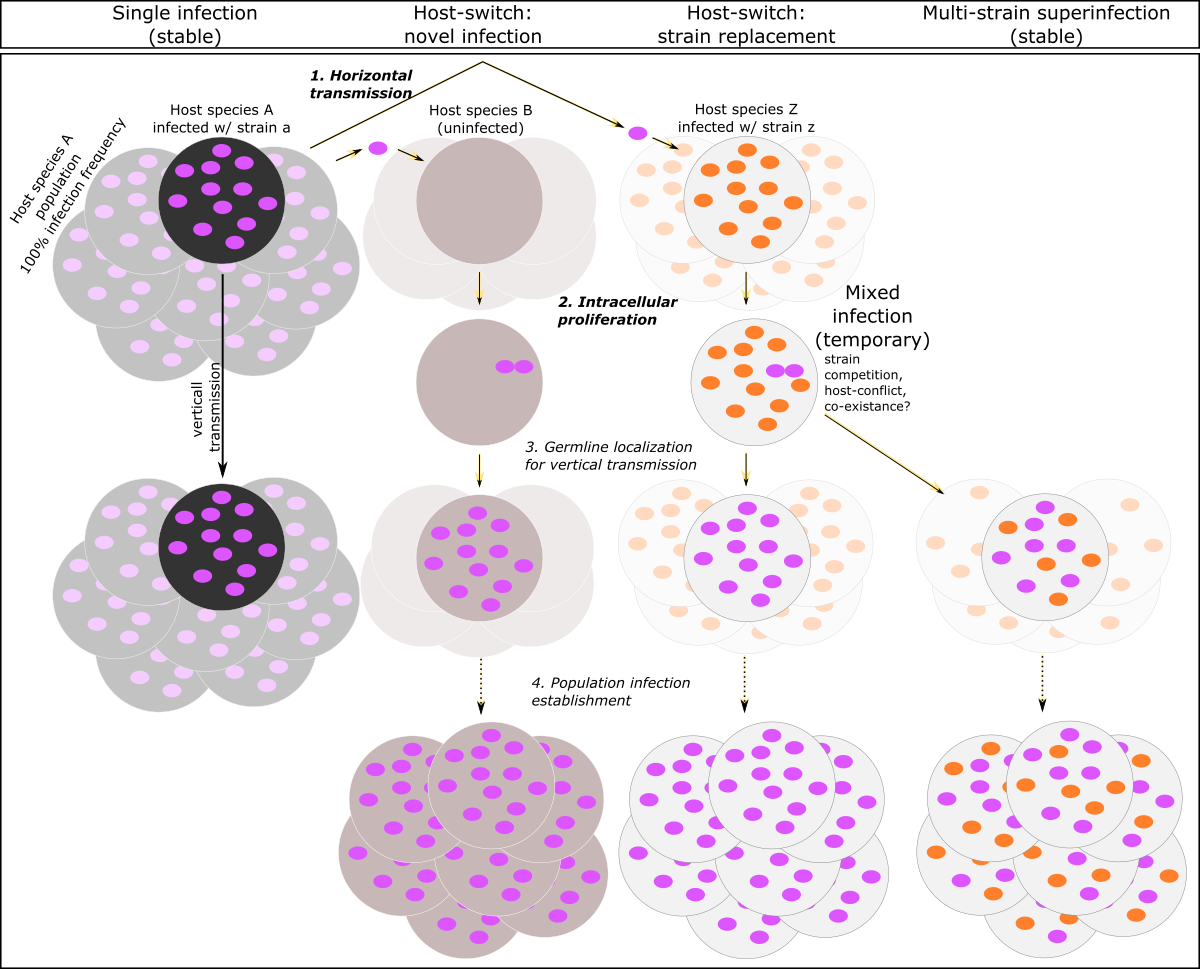

Supplement: S1 Fig — Host-switching of an endosymbiont requires successful horizontal transmission, intracellular proliferation, germline targeting for vertical transmission, and a mechanism for population establishment. Here, we use an in vitro Wolbachia-infected cell culture system to study the early stages in this process (#1 and 2 in bold) that are often lost to chance. By focusing on closely related strains with promiscuous and stable host-associations, we can understand how cell identities, divergent hosts, and resident strains impact novel infection events. (TIF) [file ppat.1012149.s001.tif]

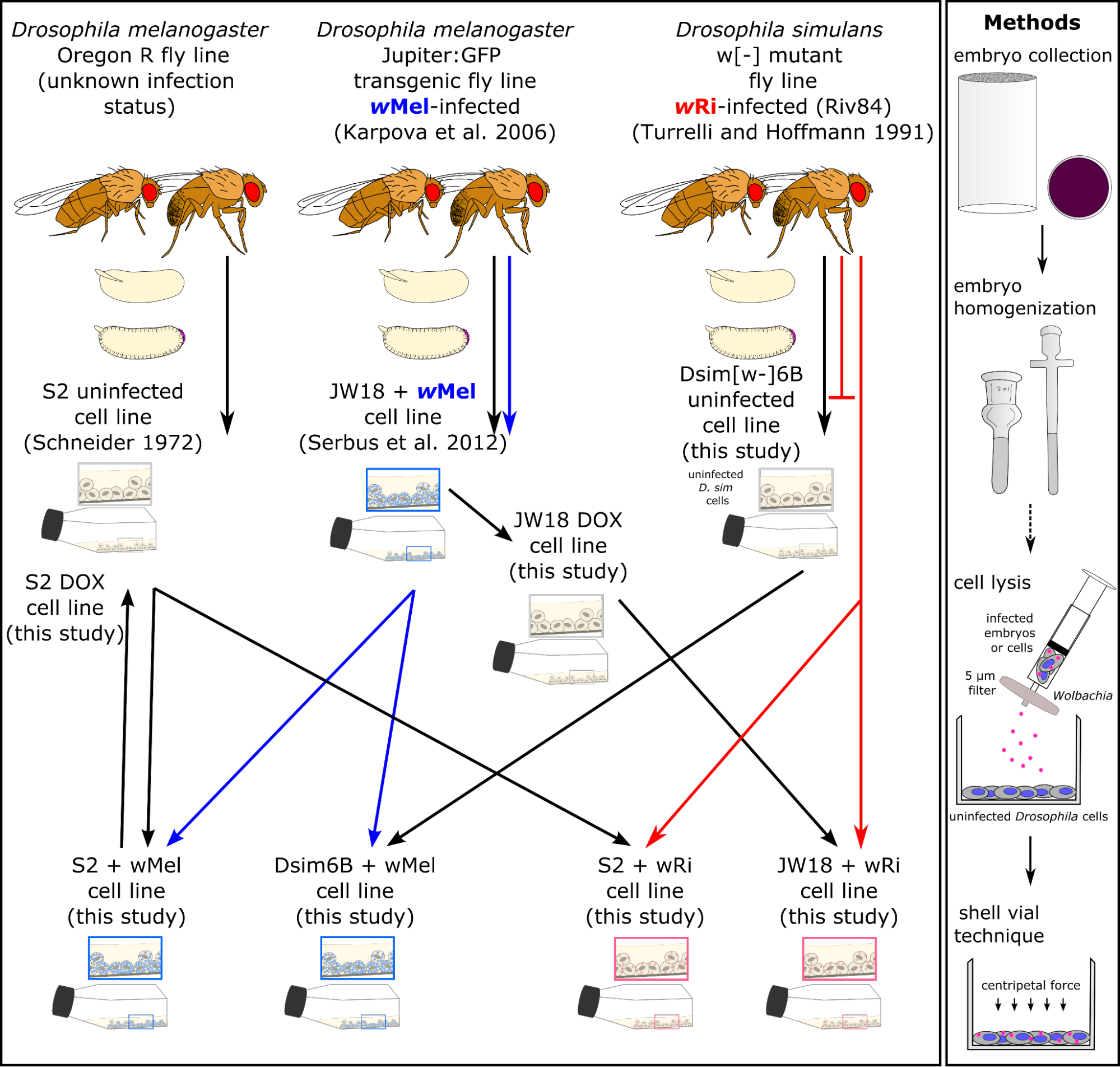

Supplement: S2 Fig — The S2 and JW18 D. melanogaster cell lines were derived previously from fly embryos of unknown infection status and infected with wMel, respectively. The Dsim6B cell line was derived in this work, from embryos from the D. simulans white eye fly line infected with the Riv84 wRi strain (see methods panel through embryo homogenization). Uninfected cell lines were obtained by treatment with 10 μg/mL doxycycline (DOX) in the cell culture media for nine weeks, followed by at least two months recovery from antibiotic treatment mitochondrial effects. Wolbachia strains were swapped among cell lines with the shell vial technique (see methods panel through shell vial technique). (TIF) [file ppat.1012149.s002.tif]

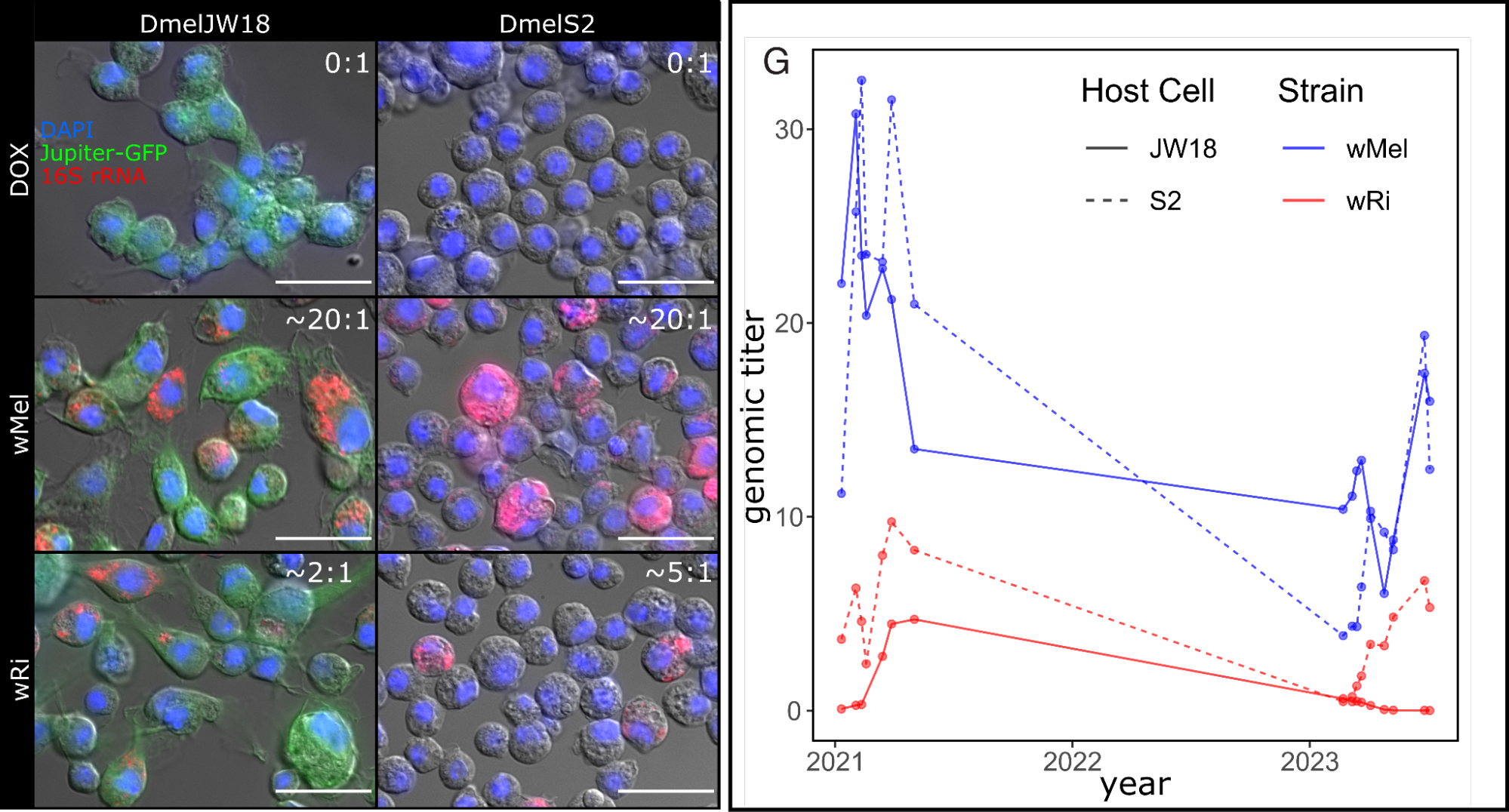

Supplement: S3 Fig — The wMel strain is consistently at ∼10x higher titer than the wRi strain in D. melanogaster cells. Titers measured in 2021 were from cells maintained at 25–26°C, whereas titers measured in 2023 were from cells maintained at 23°C. Temperature has a similar impact on both strains titers, with both exhibiting proportionately lower titers at 23°C than 25–26°C. (TIF) [file ppat.1012149.s003.tif]

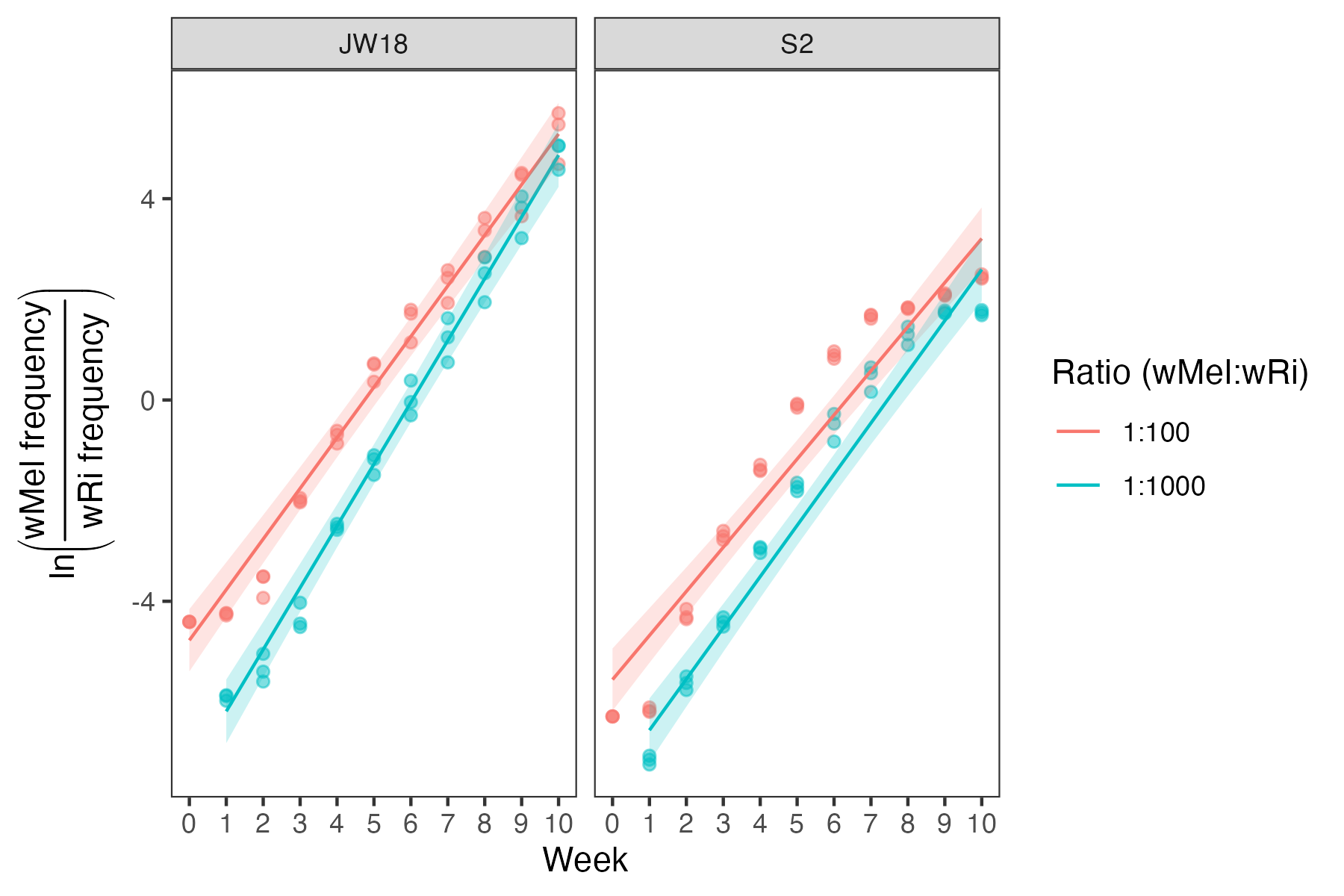

Supplement: S4 Fig — To assess how cell line and initial infection ratios influenced wMel’s competitive advantage over wRi, we utilized a linear mixed-effects model incorporating these variables as fixed effects. Prediction lines and 95% confidence intervals from the model and observed points for the two cell lines A) JW18 and B) S2 at starting ratios 1:100 (red) and 1:1000 (blue). (TIF) [file ppat.1012149.s004.tif]

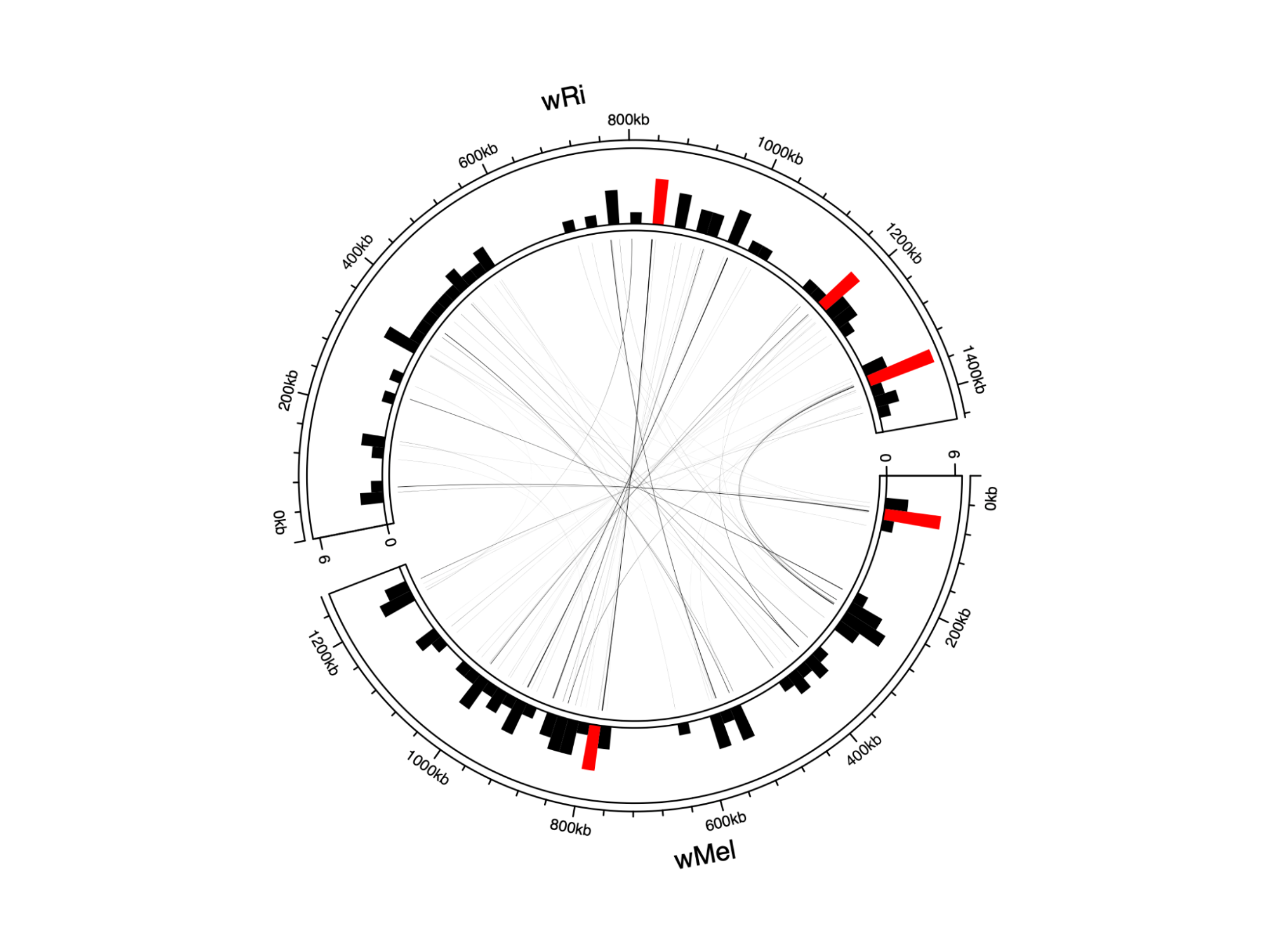

Supplement: S5 Fig — Recombinant alignments were detected by extracting the reads chimerically mapped to the wMel and wRi genomes in regions of high mappability (containing SNPs, indels, or structural variation). (TIF) [file ppat.1012149.s005.tif]

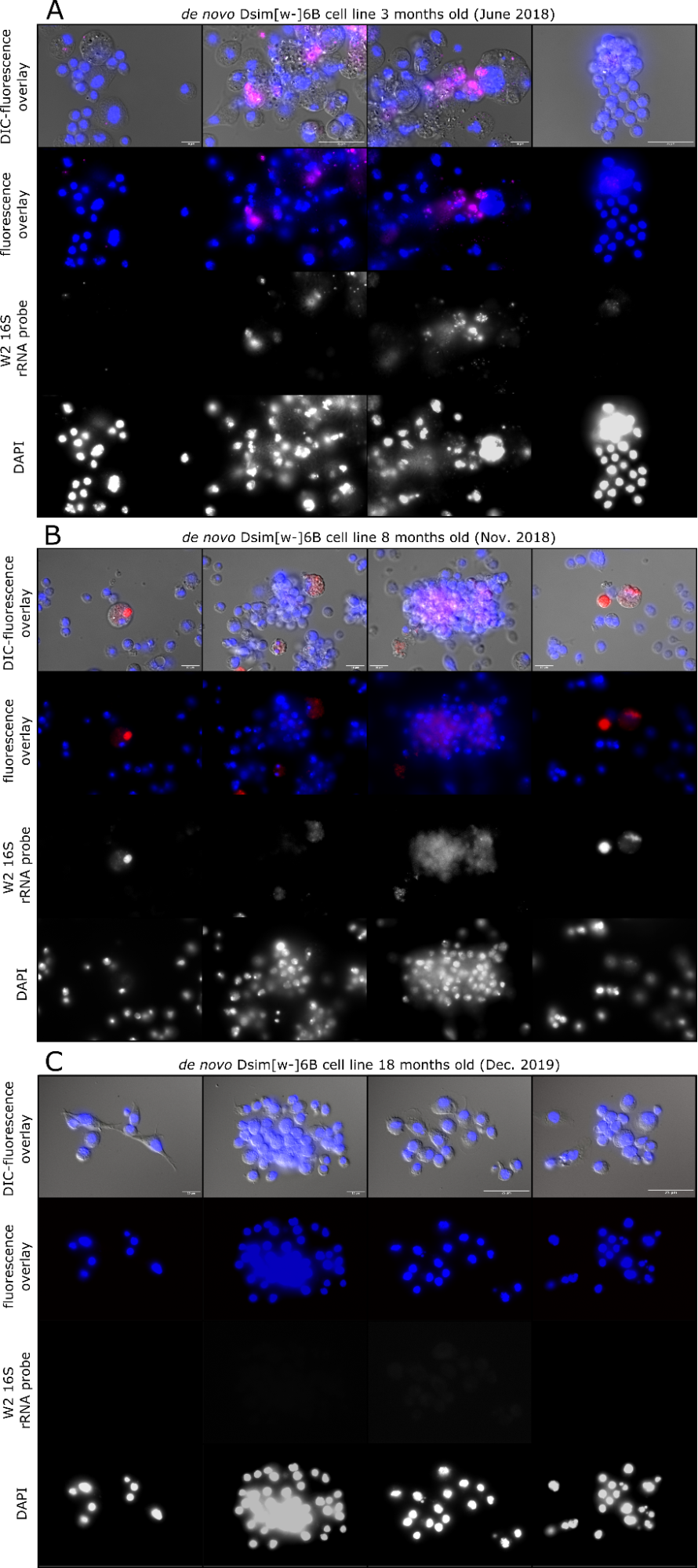

Supplement: S6 Fig — (TIF) [file ppat.1012149.s006.tif]

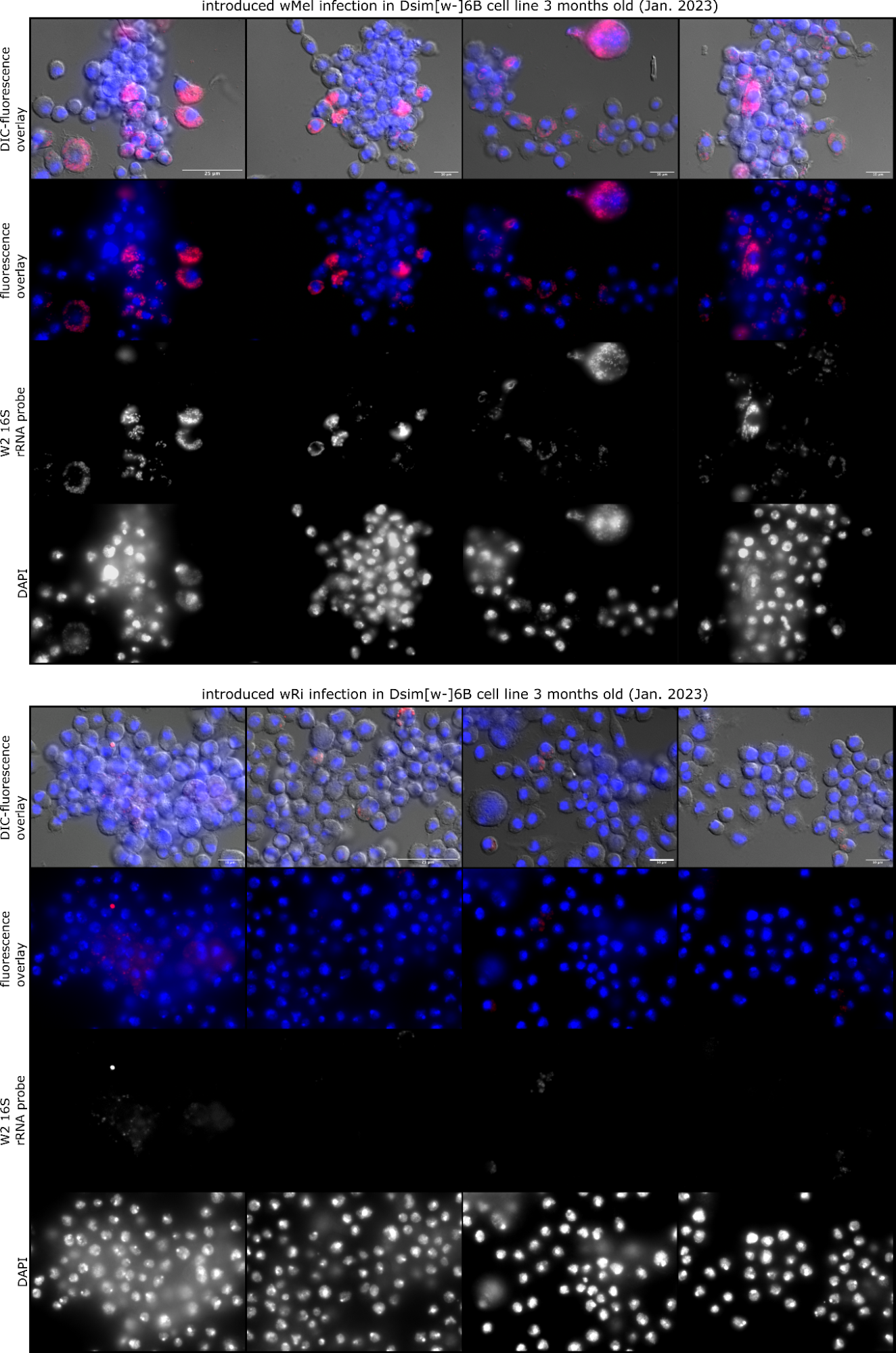

Supplement: S7 Fig — wMel (top) and wRi (bottom) strains of Wolbachia. (TIF) [file ppat.1012149.s007.tif]

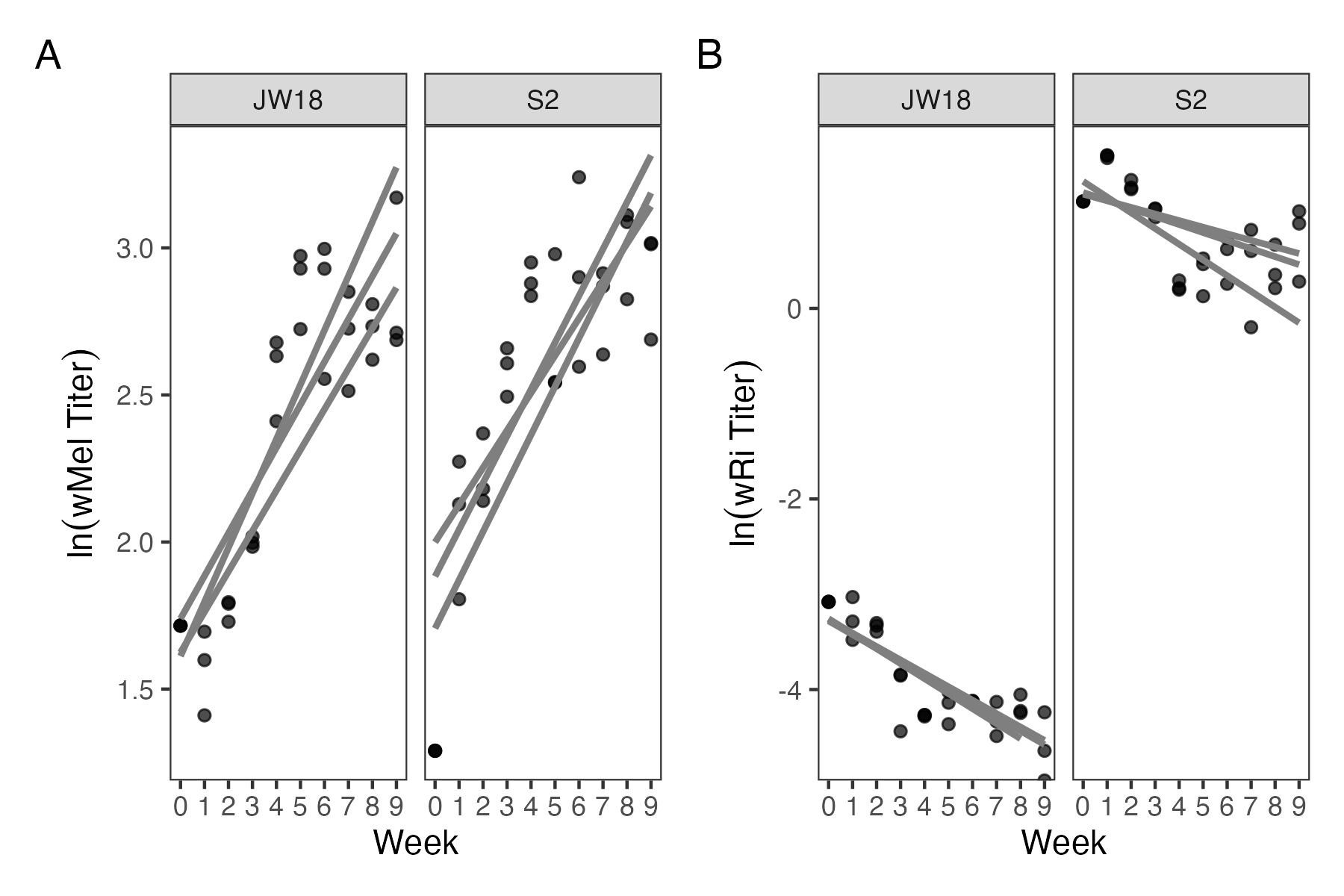

Supplement: S8 Fig — Log-linear regression analysis for A) wMel and B) wRi in 1:1 mixtures with uninfected cells. Regression summary statistics are annotated in S3 Table. (TIF) [file ppat.1012149.s008.tif]

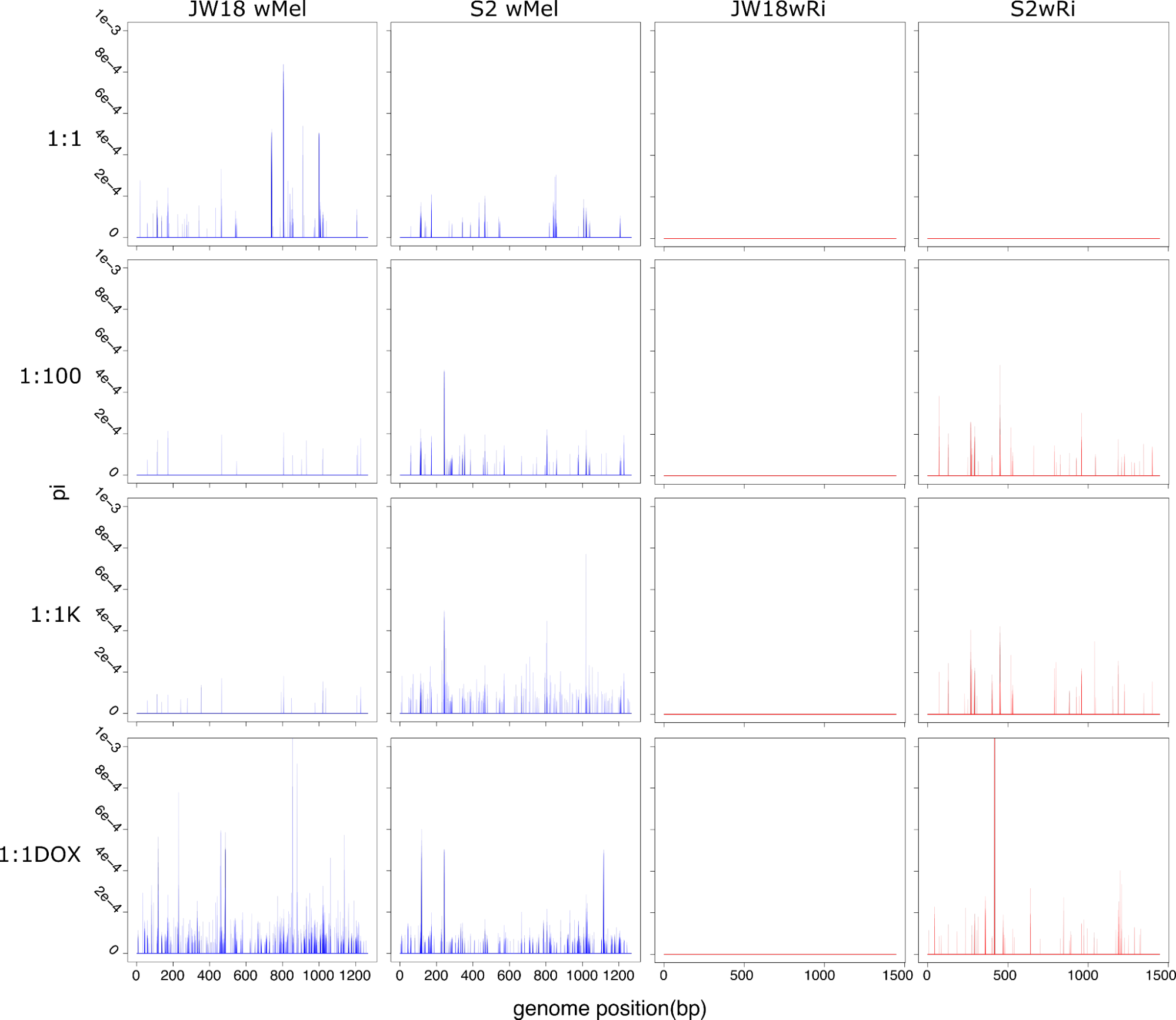

Supplement: S9 Fig — Less wRi allelic variation was detectable than for wMel because of wRi’s relatively low titer in the JW18 cell line. (TIF) [file ppat.1012149.s009.tif]

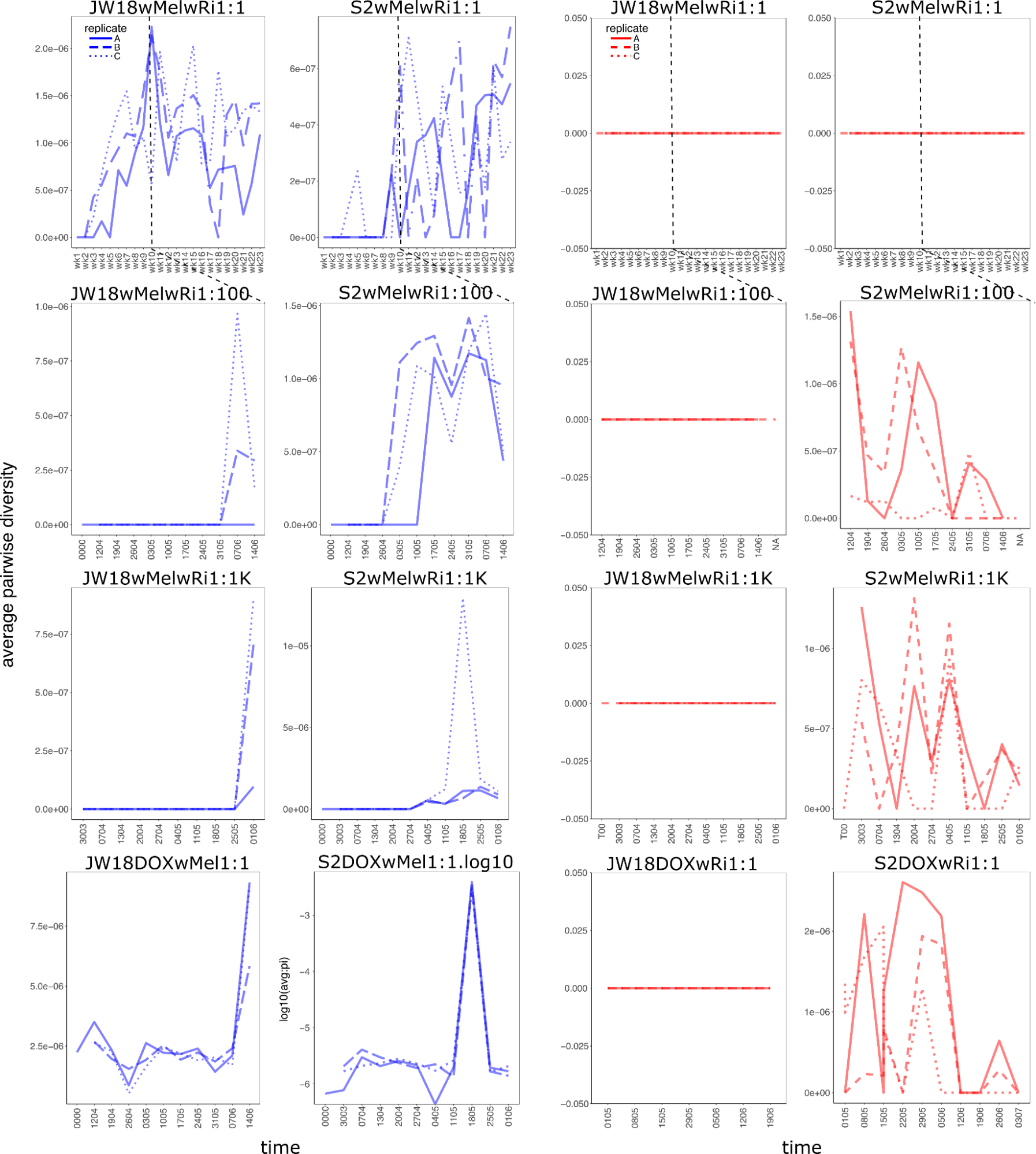

Supplement: S10 Fig — (TIF) [file ppat.1012149.s010.tif]

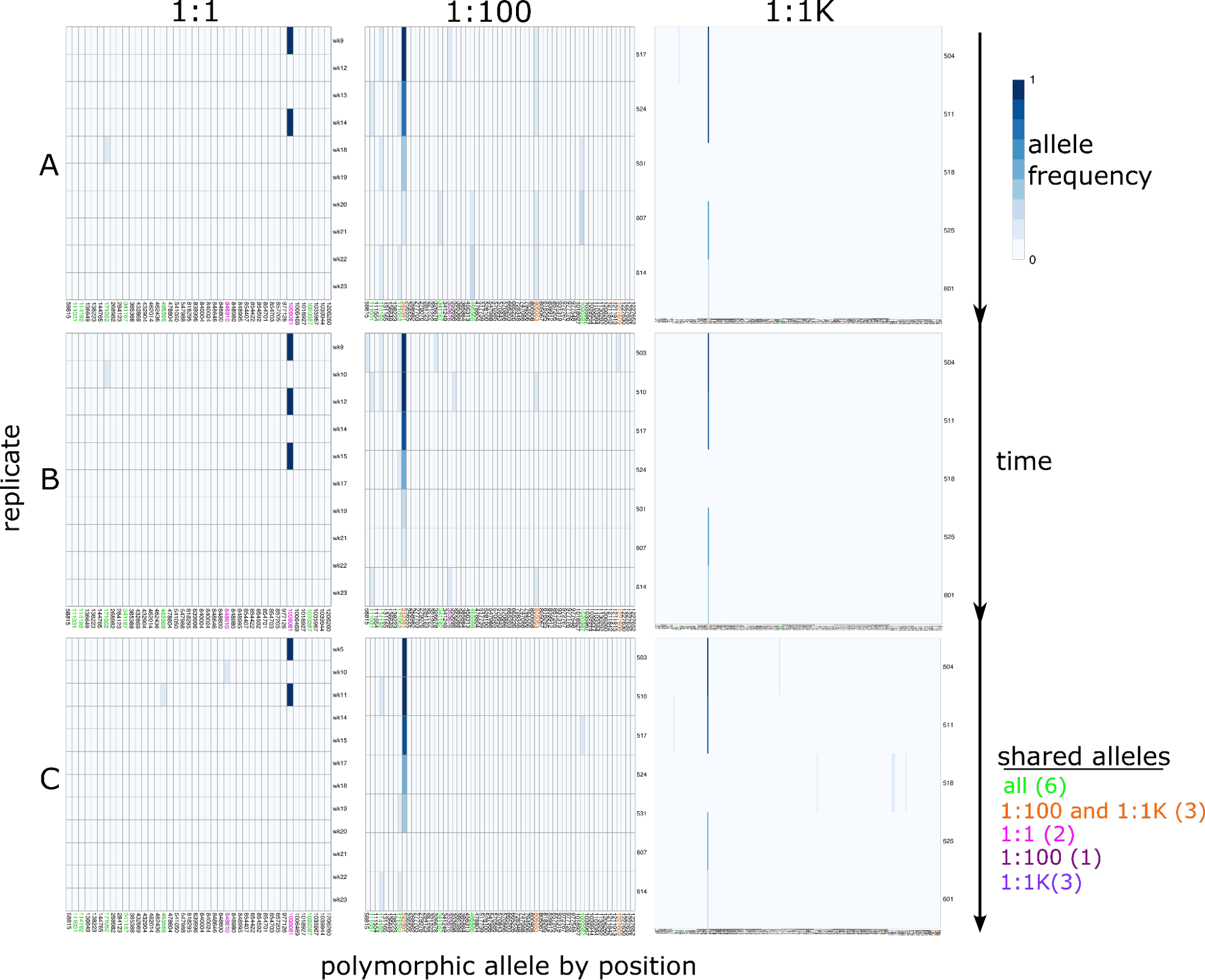

Supplement: S11 Fig — (TIF) [file ppat.1012149.s011.tif]

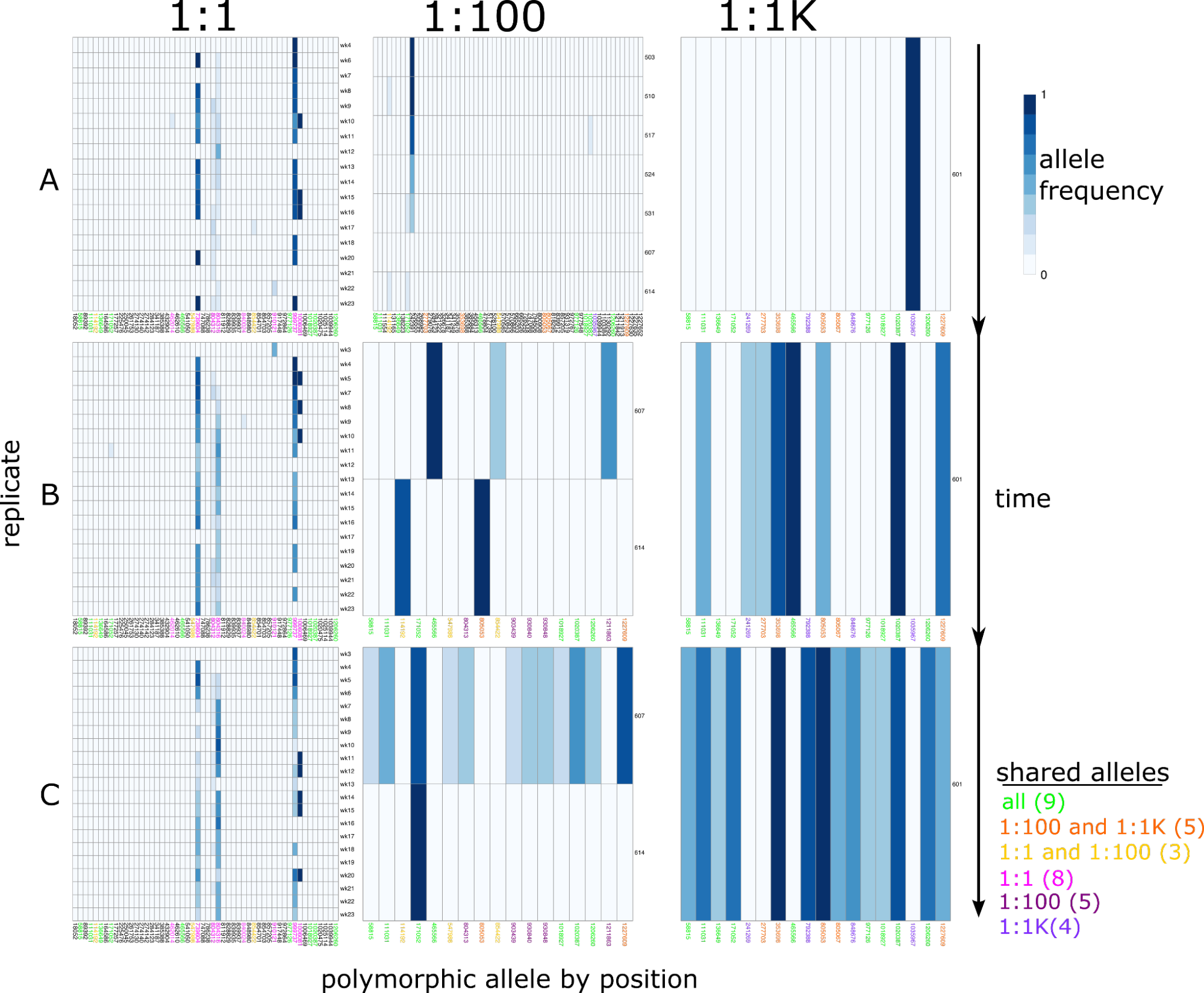

Supplement: S12 Fig — (TIF) [file ppat.1012149.s012.tif]

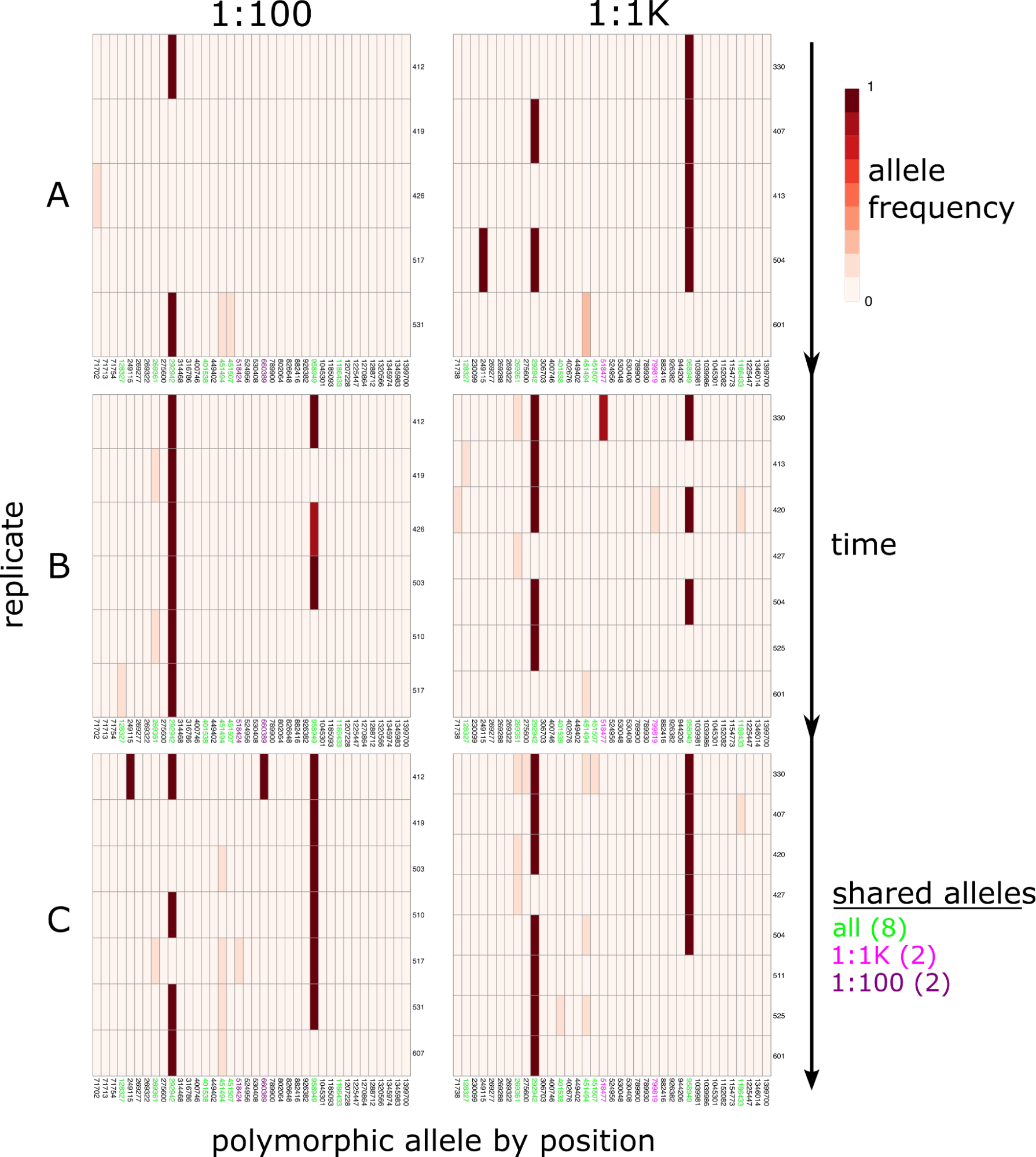

Supplement: S13 Fig — Variation in the wRi genome was not detected in the 1:1 experimental mixtures or JW18 mixtures due to the lower titer and wRi genome sequencing depth of those samples. (TIF) [file ppat.1012149.s013.tif]

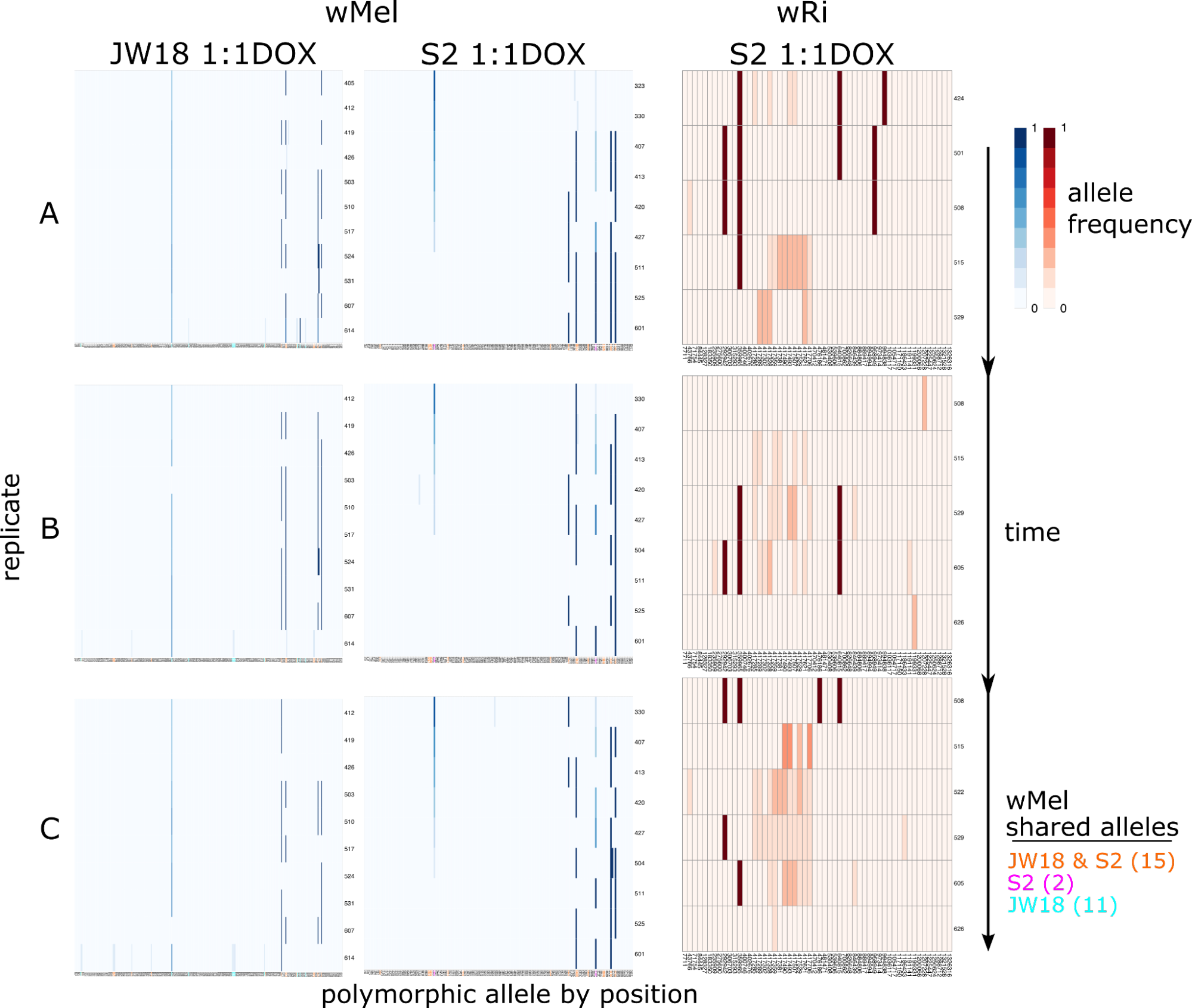

Supplement: S14 Fig — (TIF) [file ppat.1012149.s014.tif]
