# Supplementary material for: Mixed Wolbachia infections resolve rapidly during in vitro evolution
Source: PLoS Pathog. 2024 Jul 25;20(7):e1012149. doi: 10.1371/journal.ppat.1012149 (PMC11302900; doi:10.1371/journal.ppat.1012149)
Supplement: Attachment — Submitted filename: R2R.pdf [file ppat.1012149.s023.pdf]

UNIVERSITY OF CALIFORNIA, SANTA CRUZ

BERKELEY • DAVIS • IRVINE • LOS ANGELES • MERCED • RIVERSIDE • SAN DIEGO • SAN FRANCISCO SANTA BARBARA • SANTA CRUZ

Dear Elizabeth,

Thank you very much for your comments on our manuscript and for the helpful reviews. We took care to address concerns regarding *in vivo* interpretations from this *in vitro* work. Indeed, the unique attributes of *in vitro* systems enable us to study *Wolbachia* proliferation and impact on host cellular growth separately from host cellular development. We never intended to imply that our *in vitro* system was a perfect proxy for *in vivo* phenomena.

We addressed the reviewer comments below, with each comment printed in blue italics, in its entirety. Our responses are below each comment, with edited text from the manuscript in quotes. We appreciate the reviewers' thorough attention to detail. We believe their suggestions have improved the manuscript and we thanked them in the acknowledgements.

Kind regards,

Shelbi Russell
Assistant Professor, Department of Biomolecular Engineering
University of California Santa Cruz, 1156 High St. Santa Cruz, CA 95064

Dear Mirchandani,

Thank you very much for submitting your manuscript "Mixed Wolbachia infections resolve rapidly during in vitro evolution" for consideration at PLOS Pathogens. As with all papers reviewed by the journal, your manuscript was reviewed by members of the editorial board and by several independent reviewers. The reviewers appreciated the attention to an important topic. Based on the reviews, we are likely to accept this manuscript for publication, providing that you modify the manuscript according to the review recommendations.

Please be sure to address all concerns Reviewer 1 in particular raises very valid concerns about over interpretation given that the work is in cell culture.

Please prepare and submit your revised manuscript within 30 days. If you anticipate any delay, please let us know the expected resubmission date by replying to this email.

When you are ready to resubmit, please upload the following:

[1] A letter containing a detailed list of your responses to all review comments, and a description of the changes you have made in the manuscript.

Please note while forming your response, if your article is accepted, you may have the opportunity to make the peer review history publicly available. The record will include editor decision letters (with reviews) and your responses to reviewer comments. If eligible, we will contact you to opt in or out

[2] Two versions of the revised manuscript: one with either highlights or tracked changes denoting where the text has been changed; the other a clean version (uploaded as the manuscript file).

Important additional instructions are given below your reviewer comments.

Thank you again for your submission to our journal. We hope that our editorial process has been constructive so far, and we welcome your feedback at any time. Please don't hesitate to contact us if you have any questions or comments.

Sincerely,

Elizabeth A McGraw, PhD

Academic Editor

PLOS Pathogens

Debra Bessen

Section Editor

PLOS Pathogens

Michael Malim

Editor-in-Chief

PLOS Pathogens

orcid.org/0000-0002-7699-2064

****

Reviewer Comments (if any, and for reference):

Reviewer's Responses to Questions

Part I - Summary

Please use this section to discuss strengths/weaknesses of study, novelty/significance, general execution and scholarship.

Reviewer #1: This work addresses an interesting question in the field - how is it that different Wolbachia strains proliferate within and between populations? What happens during co-infections? Co-infections do exist in the field and are often between unrelated Wolbachia strains (A and B type). This may be an artifact of detection (MLST differences) and it is likely based on recent work that Wolbachia are actually a cloud of genetic variants within a single host (see Chu et al https://onlinelibrary.wiley.com/doi/full/10.1111/1744-7917.12566).

Regardless, here the authors try and use cell culture to quantify a strain's ability to persist in a host population. There are some major caveats to this approach that I do not believe the authors fully appreciate given their interpretations. That said, I wouldn't throw out the baby with the bath water as there are some very interesting observations here (especially the conclusion that recombination between strains is frequent enough to observe in the lab - Figure S5).

Reviewer #2: Summary: The paper studies what happens when wMel and wRi strains infect the same cell culture. wMel outcompetes wRi quickly and consistently. Notably the authors contribute a novel cell culture line of D.sim. Overall they setup a model of wolbachia cell-to-cell invasion which is quite interesting. In general, the paper is well written. The experimenters do a good job of planning out the next most logical experiments and so the paper has good flow and makes complete sense. The analysis is sharpened by the selection coefficients. Overall, it seems the real importance of this study is in setting up a quantifiably assay that can dissect competitive wolbachia cell-to-cell invasion and colonization, which is an interesting tool. Long-term this research could pave the way toward dissecting mechanisms that might make wolbachia more permissive to new hosts, which could be a great biotechnological help/tool. This might be emphasized in the discussion.

Yes, this is definitely something we are pursuing in the cells. We added this statement to the conclusion: "*Host in vitro systems could also provide a microcosm to select or engineer Wolbachia strains to be more permissive to new hosts and new cell type tropisms, expanding the utility of Wolbachia strains as biological control tools.*"

Part II – Major Issues: Key Experiments Required for Acceptance

Please use this section to detail the key new experiments or modifications of existing experiments that should be absolutely required to validate study conclusions.

Generally, there should be no more than 3 such required experiments or major modifications for a "Major Revision" recommendation. If more than 3 experiments are necessary to validate the study conclusions, then you are encouraged to recommend "Reject".

Reviewer #1: For the experiments detailed in all the figures, my concern is that each Wolbachia strain impacts cell growth differently under these conditions. The authors clearly measure this later on in the text and come to the conclusion that wRi massively impacts the replication rate of the host cell. It is therefore not surprising, nor particularly interesting, that the wMel infected cell population would take over.

I would've liked to have seen a difference in cell to cell spread. The authors assume that Wolbachia is spreading cell to cell but do not actually test for this. An appropriate test would be to use two different FISH probes and flow sort based on signal. This would help to quantify whether what they observe is spread vs. a dominant effect on host cell replication.

Thank you for these considerations - we too would love to have a way of measuring Wolbachia's cell-to-cell spread on a fine scale. Unfortunately, there are currently no ways of labeling live Wolbachia for multiple week experiments. FISH probes require that the cells be fixed (killed), permeabilized, and incubated at high temperatures (36-42C). Even if there were a way of using the developed 16S rRNA FISH probes on living Wolbachia, the existing probes do not distinguish wMel from wRi (difficult to do with closely related 16S sequences and short probes). The live dyes that have been used for Wolbachia staining, e.g., SYTO dyes, do not persist for long enough to watch cell-to-cell transfer between differently stained Wolbachia cultures. After a couple of hours, the dyes diffuse and limit resolution. Given that SYTO staining cannot last through one day, it is unlikely to survive *Wolbachia* cell division. We are working on approaches to make transgenic GFP/RFP-labeled *Wolbachia* and antibiotic treatments to impede extracellular transfer. But, this is beyond the scope of this paper.

To understand how wMel and wRi infections spread, we performed the DOX:wMel/wRi experiments that mixed infected cells with uninfected cells (Figure 4). The cell count data show that both strains lower host cell division rates significantly, although wRi may lower JW18 cell division rates slightly more than wMel. This means that wMel's spread in DOX:wMel mixture experiments was due to cell-to-cell transfer, not excess proliferation of wMel-infected cells relative to uninfected cells. Similarly, wRi's inability to establish in DOX:wRi mixtures indicates that it was able to spread to uninfected cells by cell-to-cell transfer less well than wMel. Thus, while we cannot quantify the rate of cell-to-cell transfer vs segregation with host cell division yet, we can disentangle these parameters' effects on infection frequencies through these combinatorial experiments. We explained this in the main text and added the following clarification (in bold), but please let us know if further explanation is needed:

> *"This transfer process not only increases wMel frequency in the culture, but also prevents uninfected host cells from remaining uninfected and out-growing the infected cell population.* ***Thus, despite not currently being able to specifically label, track, and distinguish living Wolbachia strains, we were able to detect indirect evidence of wMel's superior cell-to-cell transfer ability, relative to wRi.****"*

The only alternative explanation we can identify that would be consistent with the data is that the wMel strain has some non-cell-autonomous way of inhibiting the growth of uninfected cells in cell culture, whereas wRi lacks this ability. We have no evidence of this occurring, but it has not been tested for directly. Even if this was part of the explanation, wMel is known to transfer cell-to-cell rapidly (White et al. 2017) and some transfer would be needed to explain the maintenance of cell culture cell abundances across time points. The wMel-DOX experiments did not exhibit reduced host cell growth relative to the wRi-DOX experiments.

Reviewer #2: Major comments:
One really interesting piece of data here - is that the authors seem to be able to quantify the recombination rate of 1/500,000 genomes. This is the frequency of how often wMel and wRi are recombining in the co-culture. This is very interesting to me. Where did those recombinations occur and what information was exchanged? Was it large whole swaths of genome or small patches? What genes were contained in the swapped information? more work should be visually done to extract the information from that very valuable dataset. Are the recombinants random, or do the same regions keep recombining? Can this information be extracted and presented?

Thank you for these ideas. We agree that the chimeric alignment data are very interesting and valuable. While it is difficult to confidently identify recombinant haplotypes given the sequence similarity between wMel and wRi as well as the limited resolution provided by short reads, we have expanded these analyses to identify potential recombinant events. We conservatively filtered the chimeric alignments and clustered overlapping alignments within experiments to identify putative recombination events. Details of these steps have been added to the "Recombinant haplotype detection with Illumina sequencing" sub-section of the Methods. We have replaced Figure S5 with a Circos plot showing regions of each genome connected by putative recombinant events, as well as the number of events in that region. The amended results section now reads:

"Given Wolbachia's propensity for recombination [22–24], we tested for the presence of recombinant haplotypes between the competing strain genomes in the 1:1, 1:100, and 1:1000 wMel:wRi mixed infection experiments. Putative recombinant events were detected by identifying overlapping chimeric alignments to both the wMel and wRi genomes in regions of high mappability. In total, we identified 67 recombinant events across all experiments. Although these events were extremely rare, we found they were relatively well distributed across both genomes. However, we did identify enrichment of recombinants in three 20kb regions in wRi and two 20kb regions in wMel (Supplemental Fig S5). The highest number of putative recombinant events occurred when strains co-occurred the longest, in the 1:1000 S2 mixtures. (Table S6). These results make intuitive sense, as recombination mediated through passive processes such as homology-directed repair with divergent strain eDNA requires high concentrations (equal strain mixtures) and many chances (long co-culture times)."

While it would be exciting to identify what genes were exchanged in these recombinant events, due to their low frequency and the low resolution provided by short reads, we are unable to confidently determine this information. However, this would be an exciting opportunity for follow up work using long read sequencing to interrogate these questions.

Is the strain being compared wMelPopcorn? which is known to be more infectious than wMel? Somewhere, there should be a discussion on the Jw-18 cell line, and it's particular wolbachia infection, to clarify this point.

No it is not, but we do know that the octomom region has been deleted in the wMel strain we used. We clarify the source and genomic identity of the wMel strain we used in our experiments in the following statement added to the methods section:

“All wMel infections were initialized with the wMel strain that naturally immortalized in the JW18 cell line [20]. This wMel strain is known to lack the octomom region [37], which is linked to high titer in the wMelPop strain [38].”

Were there any genomic differences that emerged post competition? For example, does the sequencing data show any snps or fixations of mutations that were selected for - after the competition assay – when compared to the reads at the start of the competition assay or before? Can this information be extracted and presented?

Thank you for this suggestion. Yes, we were able to perform an intra-sample analysis to see whether the in vitro infections harbored genetic diversity and if any of this variation changed in frequency over the course of the experiments. The results are preliminary, as we did not design these experiments for this purpose and sequencing depth increased as Illumina flow cells increased in throughput. Thus, we want to couch these findings very carefully - this question is beyond the scope of the paper and will need to be addressed in a future manuscript with long-read and ultra-high-depth sequencing. We’ve added the following section to the manuscript, and associated supplemental figures and tables:

“The *in vitro Wolbachia* infections harbored low levels of genetic variation and this variation fluctuated across experimental time points. Average genome-wide pairwise diversity ranged from zero to 8.1e-05 (Fig S9). Across experimental time points, diversity increased with increasing titer (Fig S10) because detection of variation was limited by the depth of *Wolbachia* genome sequencing coverage (Table S0). Despite the low levels of variation and the limits of sequencing depth in low-titer infections and time points, we did detect a handful of *w*Mel and *w*Ri SNP and indel alleles that fluctuate in frequency, but do not reach fixation (Fig S11-S14). These alleles were shared among replicates and cell lines, suggesting that they were introduced as standing variation from the stable cell lines, opposed to being derived from *de novo* mutation in the experiments. The 15 high-frequency *w*Mel variant sites were located in hypothetical proteins, pseudogenes, ncRNAs, and protein-coding genes involved in oxidative stress and electron transfer (Table S7). The five high frequency *w*Ri variant sites were located exclusively in pseudogenes and intergenic regions. While some of these alleles may confer fitness benefits in vitro, either for competition or infection, the mutations in intergenic regions and pseudogenes are likely neutral and pose no impact on the fitness of *Wolbachia*. These results hold promise for our ability to track allelic variation under natural selection in *Wolbachia* in vitro infections. Future work will confirm the functional nature of these mutations with deep long read sequencing”

Part III – Minor Issues: Editorial and Data Presentation Modifications
Please use this section for editorial suggestions as well as relatively minor modifications of existing data that would enhance clarity.

Reviewer #1: Throughout, I would be very cautious about making interpretations about Wolbachias' behavior in whole animals - and populations - based on its behaviors in cell culture.
We completely agree, and have tried to couch our conclusions appropriately. Given that cell lineage developmental constraint is a primary difference between in vivo and in vivo systems, we think our interpretations are conservative. Please let us know if there is a statement in particular that you have an issue with.

Writing/interpretation:

“revealing differences between cellular and humoral regulation” - this statement is quite broad and does not consider the fact that the in vitro cell environment is really quite weird and does not well represent any biological system super well. As the authors know, the cells are often multinucleate and since immortalized, have all sorts of chromosomal and cellular oddities.

While we agree that the *in vitro* cell environment does not represent any biological system well, we were unable to main wRi infections in *D. simulans* cell lines, while wMel infections were successful and stable. This contrasts the stable, relatively high titer infections of wRi in *D simulans* flies; which overall suggests differences between wMel and wRi *in vitro* and *in vivo*. In order to make this more clear, we have modified the sentence to say:

“revealing differences between the two strains in cellular and humoral regulation”

I would also caution the authors about this statement below: “Our in vitro experimental framework for estimating cellular growth dynamics of Wolbachia strains in different host species, tissues, and cell types provides the first strategy for parameterizing endosymbiont and host cell biology at high resolution. This toolset will be crucial to our application of these bacteria as biological control agents in novel hosts and ecosystems.” If the authors wanted to start to answer questions about tissue tropism and growth dynamics therein, these cell lines are not the best place to begin. There is a collection of DGRC cell lines from different Drosophila melanogaster tissue types but even then, they are not the same genetic background which make complicate interpretation.

In the abstract, the statement that Wolbachia evolved after the divergence of arthropods and nematodes is not supported by any citation.

PLoS Pathogens’ format does not support citations in the abstract. This claim is supported with citations first-thing in the introduction with the statement:

“*The alphaproteobacterium Wolbachia pipientis became a widespread intracellular symbiont of arthropods and nematodes through its ability to infect novel hosts and establish germline transmission. Hundreds of millions of years after the divergence of Arthropoda and Nematoda (ca. 500 mya [1,2], Wolbachia endosymbionts evolved (ca. 100-200 mya [3]).*”

I found the use of the words “faithful” and “promiscuous” to be distracting and frankly, off-putting - why personify?

Thank you for this opinion. Do you have a better suggestion for terms? The intent was not to “personify”, but to describe their distributions among hosts. The wMel strain is faithful - it is only found in D. melanogaster in nature. Whereas, the wRi strain is found in many hosts, making the adjective “promiscuous” especially apt. We think these current terms are accurate and clear, while not anthropomorphizing (if that is your concern). If you disagree, please suggest alternatives that do not offend you.

Line 44 - "When a Wolbachia strain successfully infects a new host, it often encounters a resident strain that it must either replace or co-exist with as a superinfection." Is this really true? Since the ranges for infection are so broad and infections are not often fully penetrant, I am not sure I'd make this claim.

Thank you for the comment. Our intention was not to claim that mixed infections always result in superinfection or strain replacement, rather that observations of these phenomena provide evidence that mixed infections do occur at some rate. This is simply an enumeration of the natural options - a new strain will either infect an

uninfected or infected host. You are absolutely right that parameterizing this for an individual in a population is problematic because infections are variable across space and time. Considering that we see superinfections in nature and strain replacements, strain mixed infections and replacement must occur at some rate. We have softened the statement in the text to not off-put readers:

"*When a Wolbachia strain successfully infects a new host, it may encounter a resident strain, possibly resulting in a superinfection of both strains, or replacement of the resident strain by the new strain.*"

Reviewer #2: Minor comments:
Future ideas: transcriptomics of wolbachia during co-infections might provide insights on what genes help out compete.
This is a great idea and something our lab is working on currently. There are methodological challenges related to low titer, low bacterial mRNA abundance (relative to eukaryotic mRNA), and non-A-tailed transcripts that we are working to solve. Soon, we will publish work on *Wolbachia* in vitro transcriptomics with bulk dual-Seq and capable-Seq approaches. Based on our preliminary results, it will be exceedingly challenging to simultaneously sample *Wolbachia* and host transcriptomes when titer is below ~10x bacteria per cell, but capable-Seq pulldown of bacterial transcripts is more promising.

I think I understand that the competition results are not specific to only d.mel hosts because in D.sim cell lines wRi is lost, whereas wMel can be maintained stably. Is that a correct read on the data?

Yes, that is absolutely correct and a point we hope to convey in the paper. The wRi strain is a poor *in vitro* cellular symbiont in both D. melanogaster and its native host, D. simulans. Its ability to spread among hosts in nature is likely related to developmental constraints that prevent the loss of infected cell lineages during embryogenesis.

Another interesting finding is that Wolbachia slows doubling time of D.mel cells. Didn't Frydman find and publish that Wolbachia increase stem cell proliferation? can you comment on these paradoxes, is it just a fluke of different cell types? https://pubmed.ncbi.nlm.nih.gov/22021671/
Yes, both of these points are correct. However, wMel does not increase the division rate of D. melanogaster germline stem cells (GSC; see Russell et al. 2023). Also, other labs (e.g., Meany et al. 2019) have not been able to replicate the four-fold increase in GSC division in the wMau strain from Drosophila mauritiana that Frydman's group reported in 2011. We do not think this is a paradox because wMel doesn't increase GSC division. Referencing wMau's phenotypes is problematic due to the replication issue and wMau's divergence from wMel.

Some of the text in the figures is so small its quite difficult to read.

Thank you for pointing that out. We have increased the text size in the figures where it was too small to read.

PLOS authors have the option to publish the peer review history of their article (what does this mean?). If published, this will include your full peer review and any attached files.

If you choose "no", your identity will remain anonymous but your review may still be made public.

Do you want your identity to be public for this peer review? *For information about this choice, including consent withdrawal, please see our [Privacy Policy].*

Reviewer #1: No

Reviewer #2: No

Figure Files:

While revising your submission, please upload your figure files to the Preflight Analysis and Conversion Engine (PACE) digital diagnostic tool, https://pacev2.apexcovantage.com. PACE helps ensure that figures meet PLOS requirements. To use PACE, you must first register as a user. Then, login and navigate to the UPLOAD tab, where you will find detailed instructions on how to use the tool. If you encounter any issues or have any questions when using PACE, please email us at figures@plos.org.

Data Requirements:

Please note that, as a condition of publication, PLOS' data policy requires that you make available all data used to draw the conclusions outlined in your manuscript. Data must be deposited in an appropriate repository, included within the body of the manuscript, or uploaded as supporting information. This includes all numerical values that were used to generate graphs, histograms etc.. For an example see here: http://www.plosbiology.org/article/info%3Adoi%2F10.1371%2Fjournal.pbio.1001908#s5.

Reproducibility:

To enhance the reproducibility of your results, we recommend that you deposit your laboratory protocols in protocols.io, where a protocol can be assigned its own identifier (DOI) such that it can be cited independently in the future. Additionally, PLOS ONE offers an option to publish peer-reviewed clinical study protocols. Read more information on sharing protocols at https://plos.org/protocols?utm_medium=editorial-email&utm_source=authorletters&utm_campaign=protocols

References:

Please review your reference list to ensure that it is complete and correct. If you have cited papers that have been retracted, please include the rationale for doing so in the manuscript text, or remove these references and replace them with relevant current references. Any changes to the reference list should be mentioned in the rebuttal letter that accompanies your revised manuscript. If you need to cite a retracted article, indicate the

article’s retracted status in the References list and also include a citation and full reference for the retraction notice.
