# Supplementary material for: Mixed Wolbachia infections resolve rapidly during in vitro evolution
Source: PLoS Pathog. 2024 Jul 25;20(7):e1012149. doi: 10.1371/journal.ppat.1012149 (PMC11302900; doi:10.1371/journal.ppat.1012149)
Supplement: Attachment — Submitted filename: Summary_reviewerCommentsPLOS.docx [file ppat.1012149.s022.docx]

**Summary:** The paper studies what happens when wMel and wRi strains infect the same cell culture. wMel outcompetes wRi quickly and consistently. Notably the authors contribute a novel cell culture line of D.sim. Overall they setup a model of wolbachia cell-to-cell invasion which is quite interesting. In general, the paper is well written. The experimenters do a good job of planning out the next most logical experiments and so the paper has good flow and makes complete sense. The analysis is sharpened by the selection coefficients. Overall, it seems the real importance of this study is in setting up a quantifiably assay that can dissect competitive wolbachia cell-to-cell invasion and colonization, which is an interesting tool. Long-term this research could pave the way toward dissecting mechanisms that might make wolbachia more permissive to new hosts, which could be a great biotechnological help/tool. This might be emphasized in the discussion.

**Major comments:**

One really interesting piece of data here - is that the authors seem to be able to quantify the recombination rate of 1/500,000 genomes. This is the frequency of how often wMel and wRi are recombining in the co-culture. This is very interesting to me. Where did those recombinations occur and what information was exchanged? Was it large whole swaths of genome or small patches? What genes were contained in the swapped information? more work should be visually done to extract the information from that very valuable dataset. Are the recombinants random, or do the same regions keep recombining? Can this information be extracted and presented?

Is the strain being compared wMelPopcorn? which is known to be more infectious than wMel? Somewhere, there should be a discussion on the Jw-18 cell line, and it’s particular wolbachia infection, to clarify this point.

Were there any genomic differences that emerged post competition? For example, does the sequencing data show any snps or fixations of mutations that were selected for - after the competition assay – when compared to the reads at the start of the competition assay or before? Can this information be extracted and presented?

**Minor comments:**

Future ideas: transcriptomics of wolbachia during co-infections might provide insights on what genes help out compete.

I think I understand that the competition results are not specific to only d.mel hosts because in D.sim cell lines wRi is lost, whereas wMel can be maintained stably. Is that a correct read on the data?

Another interesting finding is that Wolbachia slows doubling time of D.mel cells. Didn't Frydman find and publish that Wolbachia increase stem cell proliferation? can you comment on these paradoxes, is it just a fluke of different cell types? https://pubmed.ncbi.nlm.nih.gov/22021671/

Some of the text in the figures is so small its quite difficult to read.

line 60-61, shouldn't CI be included in this list of reasons? See Turelli's new PNAS paper on why CI is so common - because CI increases the equilibrium frequencies which make it more likely that Wolbachia can make the jump to other hosts. - Turelli, M., Katznelson, A., and Ginsberg, P. S. (2022). Why Wolbachia−induced cytoplasmic incompatibility is so common. Proc. Natl. Acad. Sci. 119:e2211637119. doi: 10.1073/pnas.2211637119

line 63. Wolbachia is not a pandemic. A pandemic refers to an infectious disease typically linked to humans. The notion of wolbachia as a pandemic is detrimental to wolbachia biocontrol, especially given the recent actual pandemic of coronaviruses. I suggest you change the word. You can easily say, the planet's most common insect symbiont - or something like that.
